# Covered Stents versus Uncovered Stents for Unresectable Malignant Biliary Strictures: A Meta-Analysis

**DOI:** 10.1155/2016/6408067

**Published:** 2016-03-16

**Authors:** Ming-Yu Chen, Jia-Wei Lin, He-Pan Zhu, Bin Zhang, Guang-Yi Jiang, Pei-Jian Yan, Xiu-Jun Cai

**Affiliations:** ^1^Department of General Surgery, Sir Run Run Shaw Hospital, College of Medicine, Zhejiang University, Hangzhou, Zhejiang 310016, China; ^2^Zhejiang University, Zhejiang 310020, China

## Abstract

*Aim*. To summarize the covered or uncovered SEMS for treatment of unresectable malignant distal biliary obstruction, comparing the stent patency, patient survival, and incidence of adverse events between the two SEMSs.* Methods*. The meta-analysis search was performed independently by two of the authors, using MEDLINE, EMBASE, OVID, and Cochrane databases on all studies between 2010 and 2015. Pooled effect was calculated using either the fixed or the random effects model.* Results*. Statistics shows that there is no difference between SEMSs in the hazard ratio for patient survival (HR 1.04; 95% CI, 0.92–1.17; *P* = 0.55) and stent patency (HR 0.87, 95% CI: 0.58 to 1.30, *P* = 0.5). However, incidence of adverse events (OR: 0.74, 95% CI: 0.57 to 0.97, *P* = 0.03) showed significant different results in the covered SEMS, with dysfunctions events (OR: 0.75, 95% CI: 0.56 to 1.00, *P* = 0.05) playing a more important role than complications (OR: 0.87, 95% CI: 0.58 to 1.30, *P* = 0.50).* Conclusions*. Covered SEMS group had lower incidence of adverse events. There is no significant difference in dysfunctions, but covered SEMS trends to be better, with no difference in stent patency, patient survival, and complications.

## 1. Introduction

Stenting has become widely accepted as the treatment of unresectable distal malignant biliary obstructions (UDMBO), since Soehendra and Reynders-Frederix [1] first introduced transpapillary biliary drainage in 1980. However, the patency and incidence of adverse events (dysfunctions and complications) have an influence on the quality of life. Stent dysfunction comprised stent migration, stent occlusion causing tumor over- and/or ingrowth, which included proximal overgrowth, distal overgrowth, proximal and distal overgrowth, ingrowth, and encrustation (sludge) [2]. It can be confirmed by subsequent radiologic studies, including computed tomography, percutaneous cholangiography, and endoscopic retrograde cholangiopancreatography, and based on lab test such as recurrent jaundice. Complication comprised hemorrhage, cholecystitis, pancreatitis retroperitoneal, perforation, and cholangitis (medical therapy), which was suspected based on clinical symptoms and signs. There are various types of stents used for the management of UDMBO: plastic stents, self-expandable metallic stent (SEMS) such as uncovered and covered stents, and recent developed bioabsorbable or biodegradable stents [3]. SEMS is considered to be the most cost-effective biliary stent for treatment of unresectable malignant biliary obstructions (UDMBO), but there is still some debate on the selection of covered or uncovered SEMS in distal duct strictures. The main disadvantage of covered SEMS is migration, because of nonembedded stent body, while the uncovered SEMSs which were embedded into the biliary wall are resistant to migration, because of their mesh structure and self-expandability. Unluckily, the tumor ingrowth (TI) via the stent mesh is the main cause of occlusion in uncovered SEMS. Although there are some randomized studies comparing covered and uncovered SEMS, the results are different according to each study. One [4] showed that covered SEMS had longer patency than uncovered SEMS; another [2, 5] revealed no statistically significant difference. Interestingly, the latest meta-analysis revealed that covered SEMS did not appear to have longer patency, and benefits brought by the two SEMSs to patients were not clear [6]. Unfortunately, the meta-analysis studies which focused on complications did not pay attention to the difference in patient survival and incidence of adverse event and dysfunction events. Therefore, it is essentially necessary to get a comprehensive understanding on the difference between uncovered and covered SEMS for the treatment of unresectable distal malignant biliary obstructions, especially stent patency, patient survival, and incidence of adverse events.

## 2. Materials and Methods

This meta-analysis adhered to the guidelines of Preferred Reporting Items for Systematic Reviews and Meta-Analyses (PRISMA).

### 2.1. Study Selection

The meta-analysis search was performed independently by two of the authors (Mr. Jiang and Mr. Yan), using MEDLINE, EMBASE, OVID, and Cochrane databases. The search was performed on all studies between 2010 and 2015 to compare covered and uncovered SEMS for unresectable distal malignant biliary obstructions. The search strategy was based on the following Medical Subject Heading terms (MeSH): “unresectable,” “malignant biliary obstructions,” “distal biliary obstructions,” “distal biliary strictures,” “covered,” “uncovered,” “self-expandable metallic stents,” and “SEMS.” Only studies on humans and in English and Chinese language were considered for inclusion. Reference lists of all retrieved articles were manually searched for additional studies.

### 2.2. Data Extraction and Conversion

Data extraction was performed independently by two reviewers (Mr. Jiang and Mr. Yan, resp.). The following parameters for each study included (1) first author, the year of publication, and the study type; (2) the number and characteristics of patients; (3) the outcome of the trials including number or incidence of adverse events (dysfunction and complication) and HR of elevated stent patency and patient survival, as well as their 95%. If available, the HRs with their 95% CIs and *P* values were collected from the original article or the corresponding E-mails. If not, we calculated HRs and their 95% confidence interval using the data of observed deaths, the data of samples in each group, or the data provided by the authors. If only Kaplan-Meier curves were available, we extracted data from the graphical survival plots and estimated the HRs. All the calculations mentioned above were based on the methods provided by Tierney et al. [14] and Parmar et al. [15].

### 2.3. Inclusion Criteria

The studies included in the meta-analysis had to fulfill the following criteria: (1) they should compare the original outcomes of covered and uncovered SEMSs for the treatment of unresectable distal malignant biliary obstructions; (2) they should report on at least stent patency, patient survival, and incidence of adverse events; (3) if dual (or multiple) studies were reported by the same institution and/or authors, only the most recent publication or the highest quality of studies was included.

### 2.4. Exclusion Criteria

The following studies were excluded: (1) those dealing with biliary malignant strictures or obstructions with second stents; (2) those using other types of stents, such as plastic stents; (3) those with no clearly reported outcomes; and (4) abstracts, letters, editorials and expert opinions, reviews without original data, case reports, and studies lacking control groups.

### 2.5. Statistical Analysis

The meta-analysis was performed using Review Manager (RevMan), version 5.3. Stent patency and patient survival were analyzed using estimation of hazard ratios (HRs) with a 95% confidence interval (95% CI). Pooled effect was calculated using either the fixed or the random effects model. The test of heterogeneity of combined HRs was carried out using Cochran's *Q* test and Higgins *I*-squared statistic. If the *I* ^2^ statistic was >50%, we considered heterogeneity to be present. If the probability of a chance occurrence was less than 5% (*P* < 0.05), all statistical data were considered significant.

## 3. Results

### 3.1. Selection of Trials

**Figure 1:**
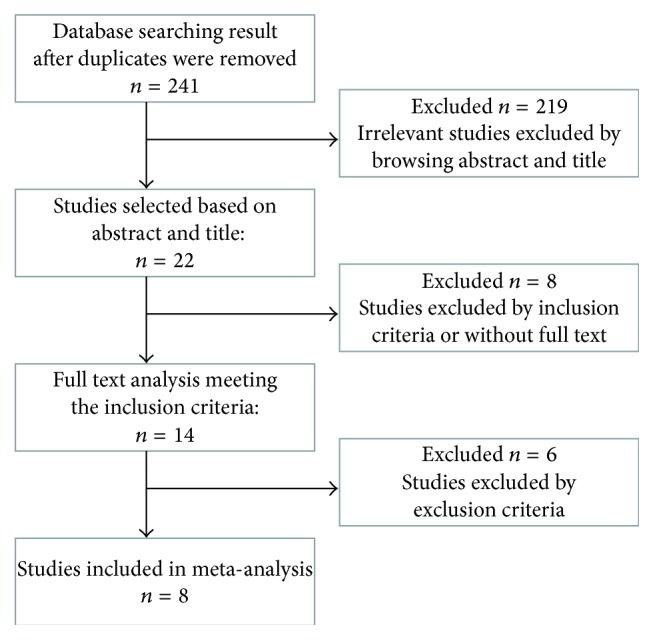
Flowchart showing selection of studies for meta-analysis.

After initial screening, of the 14 clinical trials [2, 5, 7–13, 16–20] which initially met the inclusion criteria, 2 [17, 20] did not display the specific comparison of the effects of covered and uncovered SEMS, 1 [16] used second stents, and 1 [5] dealt with patients who had received or were receiving chemotherapy, and 2 [18, 19] did not provide enough original data. Finally, 8 studies, including 2 retrospective studies and 6 prospective randomized studies, matched the selection criteria and were published between 2010 and 2015 (Figure 1). The characteristics of these 8 studies are summarized in Table 1. The 8 studies included a total of 1067 patients: 524 in the covered SEMS group and 533 in the uncovered SEMS group. The sample size of each study varied from 30 to 191 patients. The proportion of men (OR = 1.02, 95% CI: 0.80–1.31, *P* = 0.85) and the proportion of pancreatic cancer in tumor etiology (OR = 0.97, 95% CI: 0.69–1.36, *P* = 0.87) were not significant.

### 3.2. Patient Survival

**Figure 2:**
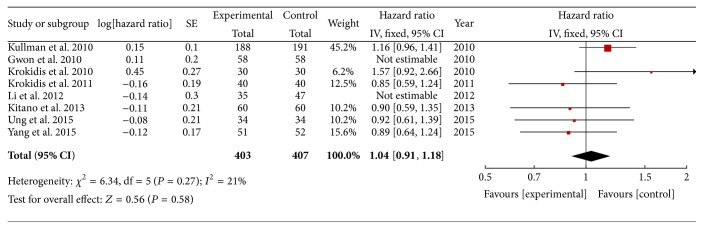
Forest plot of studies evaluating hazard ratios of patient survival.

All 8 studies reported patient survival. No statistical difference existed between the covered SEMS and uncovered SEMS in the hazard ratio of patient survival (HR 1.04; 95% CI, 0.92–1.17; *P* = 0.55). There is no heterogeneity among the 8 studies, and a fixed-effect model was used (Figure 2).

### 3.3. Stent Patency

**Figure 3:**
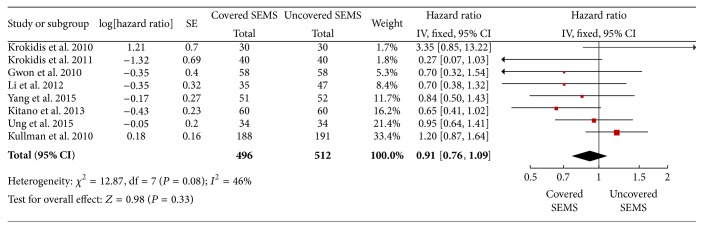
Forest plot of studies evaluating hazard ratios of stent patency.

As a fixed-effect model was used, the meta-analysis from the 8 studies also showed no significant difference of stent patency between two groups (HR 0.87, 95% CI: 0.58 to 1.30, *P* = 0.5) (Figure 3).

### 3.4. Complications and Dysfunctions Events

**Figure 4:**
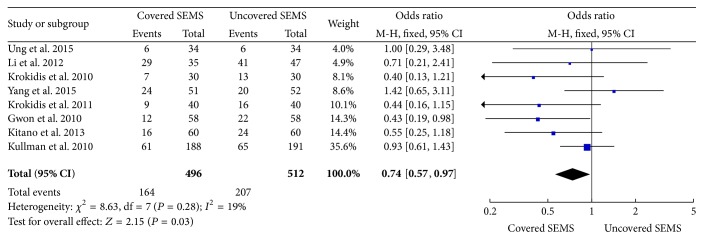
Forest plot of studies evaluating odds ratios of adverse events.

**Figure 5:**
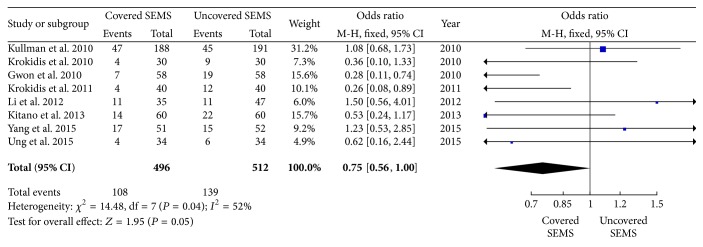
Forest plot of studies evaluating odds ratios of dysfunctions events.

**Figure 6:**
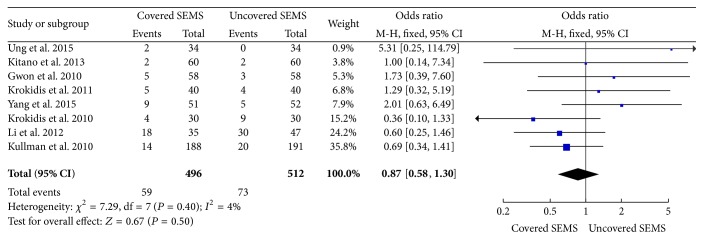
Forest plot of studies evaluating odds ratios of complications.

The statistical data was significantly favorable to covered SEMS group at incidence of adverse events (8 trials reported the data, OR: 0.74, 95% CI: 0.57 to 0.97, *P* = 0.03) (Figure 4), and there is a lower trend toward dysfunction events (8 trials reported the data, OR: 0.75, 95% CI: 0.56 to 1.00, *P* = 0.05) (Figure 5). However, there is no significant difference in complications (OR: 0.87, 95% CI: 0.58 to 1.30, *P* = 0.50) (Figure 6). According to the statistical data, the incidence of adverse events is lower in the covered SEMS, while there is no difference in complications.

### 3.5. Publication Bias

The publication bias of included studies was evaluated by funnel plots. As is shown in Figure 8, the funnel plots were almost symmetric. Hence, there was no evidence for significant publication bias in our meta-analysis.

## 4. Discussion

This meta-analysis shows that the covered SEMS group had lower incidence of adverse events (dysfunctions and complications) than the uncovered SEMS treatment group for unresectable distal malignant biliary obstructions. The main cause may be the higher reobstruction rates, which result from the tumor ingrowth and overgrowth after uncovered SEMS replacement. Although migration can contribute to the covered SEMS reobstruction or dysfunctions, the incidence may be lower than tumor ingrowth or overgrowth rate after uncovered SEMS replacement. Moreover, although the incidence of complications was not significantly different between covered and uncovered SEMS, a trend was shown in the rates of dysfunctions (OR: 0.75, 95% CI: 0.56 to 1.00, *P* = 0.05). This may explain better outcomes using covered SEMS.

Our results also suggest that covered SEMS should not provide a significant prolongation in cumulative stent patency when compared with uncovered SEMS. Although stent dysfunction had a trend towards being lower in covered SEMS at a given time, the stent patency was influenced by various factors, such as structural properties of SEMSs and presence of covering materials [21]. However, in the 8 trials, the follow-up end point was either last follow-up or patient death, and there was no difference in patient survival.

Acute pancreatitis and acute cholecystitis are the potential of complications, when the covered SEMS is placed over the cystic or pancreatic duct orifice. Although not proven in clinical studies, it has been claimed that covered SEMS might increase the prevalence of pancreatitis or cholecystitis by blocking the cystic duct orifice or pancreatic duct orifice. However, in our studies, we cannot come to the conclusion that the rate of complications was significant. It is probably due to the high portion of pancreatic cancer patients in the two groups. Additionally, this observation is commonly confounded by a low event rate [22]. What is more, such measures or precision may not be used widely in clinical practice in some hospitals. Therefore, it must be noted that measures were taken to reduce the rate of complications, either by using covered SEMSs with transmural drainage holes [8, 9] or by ensuring that the stent covering was placed below the level of the cystic or pancreatic duct.

**Figure 7:**
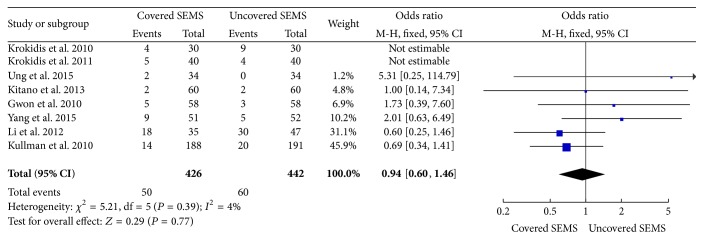
Forest plot of studies evaluating odds ratios of complications with endoscopic stent placement.

**Figure 8:**
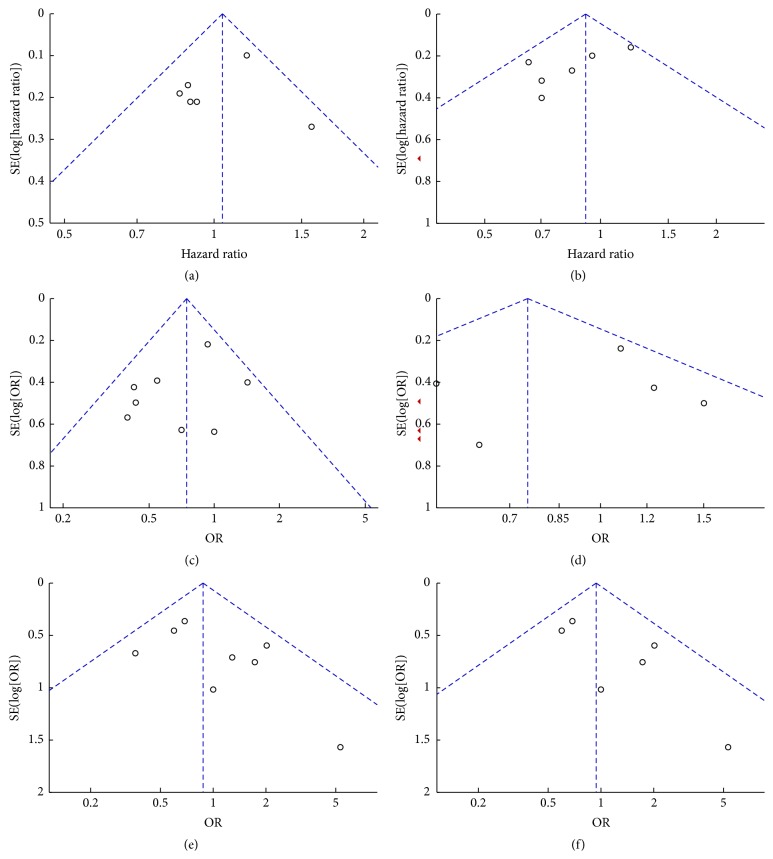
Funnel plot of studies included in the six meta-analyses: (a) patient survival, (b) stent patency, (c) adverse events, (d) dysfunctions events, (e) complications, and (f) complications with endoscopic stent placement.

In patients with unresectable malignant distal biliary obstruction, palliation may be offered with stent placement (either endoscopic or percutaneous). Endoscopic biliary stent placement seems to be associated with lower complication rate and better quality of life [23]. Therefore, endoscopic biliary stent placement is considered the gold standard, in order to improve success rates and patient outcomes. However, some studies [8, 9] we selected in this meta-analysis included percutaneous biliary stent placement, although ERCP was the initial approach, and PTC was performed when ERCP was not feasible. We cannot know how many participate in percutaneous biliary stent placement from the original article, but we get the same result in evaluating odds ratios of complications with endoscopic stent placement (OR: 0.94, 95% CI: 0.60 to 1.46, *P* = 0.39) (Figure 7).

### 4.1. Limitations and Strengths

Limitations of our analysis include the use of various covered SEMSs; ePTFE/FEP was used in two studies, whereas other materials (polyurethane, polycarbonate-polyurethane, and Permalume) were used in the remaining studies. Therefore, we assumed there were no differences in different types of covered SEMSs, like some published trials [4, 16, 24, 25].

The strength of this meta-analysis comes from the high methodological quality of each individual study as well as data homogeneity for most outcomes, including the primary outcome of stent patency, patient survival, and incidence of adverse events (dysfunctions and complications).

## 5. Conclusions

Covered SEMS group had lower incidence of adverse events (dysfunctions and complications) than the uncovered SEMS group. There is no significant difference in dysfunctions, but covered SEMS trends to be better, and there is no difference in stent patency, patient survival, and complications. Compared with uncovered SEMS, covered SEMS is recommended for treating patients who are diagnosed as having unresectable distal malignant biliary obstructions.

## Figures and Tables

**Table 1:** Baseline characteristics of studies included in the meta-analysis.

Author/(yr)	Type	Group	Covering material	Number	M/F	Mean age (yr)	Tumor etiology					
							Pancreatic cancer *n* (%)	Cholangiocarcinoma *n* (%)	Ampullary cancer *n* (%)	Gallbladder cancer *n* (%)	Metastatic lymph nodes *n* (%)	Unknown *n* (%)
Kullman et al. 2010 [2]	RCT	CSEMS	Polycarbonate-polyurethane	188	88/112	79.0 (39–100)	152 (76)	12 (6)	8 (4)	8 (3)	16 (8)	4 (2)
		UCSEMS		191	91/109	76.0 (51–95)	155 (77)	10 (5)	9 (4)	3 (2)	18 (9)	5 (3)
Gwon et al. 2010 [7]	NRCT	CSEMS	PTFE	58	37/21	63.8 (35–84)	18 (31.0)	11 (19.0)	—	6 (10.3)	19 (32.8)	4 (6.9)
		UCSEMS		58	39/19	62.7 (24–86)	17 (29.3)	16 (27.6)	—	5 (8.6)	19 (32.8)	1 (1.7)
Krokidis et al. 2010 [8]	RCT	CSEMS	Viabil	30	20/10	66.5 (52–78)	—	30 (100)	—	—	—	—
		UCSEMS		30	16/14	63.7 (46–73)	—	30 (100)	—	—	—	—
Krokidis et al. 2011 [9]	RCT	CSEMS	ePTFE/FEP	40	17/23	65.0 ± 8.8	35 (87.5)	—	—	—	—	—
		UCSEMS		40	36/4	63.5 ± 9.8	32 (80)	—	—	—	—	—
Li et al. 2012 [10]	NRCT	CSEMS	ePTFE	35	20/15	64.4 ± 11.3	5 (14.3)	22 (62.9)	5 (14.3)	1 (5.9)	—	2 (2.6)
		UCSEMS		47	30/17	63.2 ± 11.5	9 (19.1)	31 (65.9)	6 (12.8)	0 (0.0)	—	1 (2.2)
Kitano et al. 2013 [11]	RCT	CSEMS	Antimigration	60	25/35	70.6 ± 10.7	60 (100)	—	—	—	—	—
		UCSEMS		60	29/31	68.7 ± 8.9	60 (100)	—	—	—	—	
Ung et al. 2015 [12]	RCT	CSEMS	Hanaro	34	18/16	77.0 (54–88)	30 (88)	—	1 (3)	2 (6)	—	2 (5.9)
		UCSEMS		34	9/15	79.0 (54–92)	27 (79)	—	3 (9)	5 (15)	—	1 (2.9)
Yang et al. 2015 [13]	RCT	CSEMS	Silicone	51	34/17	68.7 ± 11.2	29 (56.9)	17 (33.3)	2 (3.9)	2 (3.9)	1 (2)	—
		UCSEMS		52	30/22	68.0 ± 11.3	36 (69.2)	7 (13.5)	2 (3.8)	5 (9.6)	2 (3.8)	—

RCT: randomized control trial; NRCT: nonrandomized control trial; CSEMS: covered self-expandable metallic stents; UCSEMS: uncovered self-expandable metallic stents; M: male; F: female.
